# Targeted Capture of Chinese Hamster Ovary Host Cell Proteins: Peptide Ligand Discovery

**DOI:** 10.3390/ijms20071729

**Published:** 2019-04-08

**Authors:** R. Ashton Lavoie, Alice di Fazio, R. Kevin Blackburn, Michael B. Goshe, Ruben G. Carbonell, Stefano Menegatti

**Affiliations:** 1 Department of Chemical and Biomolecular Engineering, North Carolina State University, Raleigh, NC 27695-7905, USA; rabradl3@ncsu.edu (R.A.L.); abdifazi@ncsu.edu (A.d.F.); 2 Department of Molecular and Structural Biochemistry, North Carolina State University, Raleigh, NC 27695-7622, USA; Kevin_Blackburn@waters.com (R.K.B.); mbgoshe@ncsu.edu (M.B.G.); 3 Biomanufacturing Training and Education Center (BTEC), North Carolina State University, Raleigh, NC 27695-7905, USA; 4 National Institute for Innovation in Manufacturing Biopharmaceuticals (NIIMBL), Newark, DE 19711, USA

**Keywords:** therapeutic antibodies, host cell proteins, protein purification, peptide ligands

## Abstract

The growing integration of quality-by-design (QbD) concepts in biomanufacturing calls for a detailed and quantitative knowledge of the profile of impurities and their impact on the product safety and efficacy. Particularly valuable is the determination of the residual level of host cell proteins (HCPs) secreted, together with the product of interest, by the recombinant cells utilized for production. Though often referred to as a single impurity, HCPs comprise a variety of species with diverse abundance, size, function, and composition. The clearance of these impurities is a complex issue due to their cell line to cell line, product-to-product, and batch-to-batch variations. Improvements in HCP monitoring through proteomic-based methods have led to identification of a subset of “problematic” HCPs that are particularly challenging to remove, both at the product capture and product polishing steps, and compromise product stability and safety even at trace concentrations. This paper describes the development of synthetic peptide ligands capable of capturing a broad spectrum of Chinese hamster ovary (CHO) HCPs with a combination of peptide species that allow for advanced mixed-mode binding. Solid phase peptide libraries were screened for identification and characterization of peptides that capture CHO HCPs while showing minimal binding of human IgG, utilized here as a model product. Tetrameric and hexameric ligands featuring either multipolar or hydrophobic/positive amino acid compositions were found to be the most effective. Tetrameric multipolar ligands exhibited the highest targeted binding ratio (ratio of HCP clearance over IgG loss), more than double that of commercial mixed-mode and anion exchange resins utilized by industry for IgG polishing. All peptide resins tested showed preferential binding to HCPs compared to IgG, indicating potential uses in flow-through mode or weak-partitioning-mode chromatography.

## 1. Introduction

The effective removal of host cell proteins (HCPs) from mammalian cell culture supernatants is a crucial issue in the manufacturing of biopharmaceuticals for human therapy. While general guidelines point to a maximum allowable HCP content in the final formulation of a biotherapeutic at <100 ppm [1], this measure does not fully capture the complexity of the residual HCP profile and its potential impact on a patient’s health. Concerns regarding HCP impurities stem from a variety of risk factors, including (i) an immunogenic response from repeated exposure to low concentration HCPs present in drugs administered over time [2], (ii) reduced therapeutic efficacy caused by product-bound impurities [3], and (iii) low shelf-life of the therapeutic product caused by low-concentration proteolytic enzymes present in the final formulation [4]. Current biomanufacturing practice requires the removal of 3 to 5 logs of HCP for antibody production, where the initial HCP content in the cell culture fluid is on the order of 100 mg HCP/g product. The wide variety in composition, abundance, structure, and function of HCPs, together with the variability between cell lines, processes, and production batches, makes their capture a critical challenge [5,6,7,8].

In the manufacturing of therapeutic monoclonal antibodies (mAbs), the majority of HCP impurities (>90%) [9] is removed at the product capture step, which currently relies on either affinity chromatography, mainly Protein A [10,11], or alternatively ion exchange [12] or multimodal chromatography [13]. This step is performed in bind-and-elute mode, where the mAb product is retained and non-binding impurities flow through, thus achieving both impurity removal and concentration of the target product. Process and product impurities not removed in the capture step are typically cleared in subsequent polishing steps, operated either in bind-and-elute or flow-through modes. In flow-through mode, the adsorbent features a broad specificity to allow for the capture of not only the HCP species, but also other impurities and contaminants such as DNA, leached Protein A, and media components. A number of “problematic” HCPs have been identified in the context of mAb polishing, including (i) product-bound species (e.g., nidogen-1, secreted protein acidic and cysteine-rich (SPARC) protein, clusterin) [14,15,16]; (ii) species that co-elute with mAb from protein A (e.g., histone) [3,17]; (iii) proteins that affect product stability (e.g., lipoprotein lipase) [18,19]; and (iv) species that stimulate an immunogenic response (e.g., phospholipase B-like protein) [2,20,21]. Demonstrated stability concerns generated by lipoprotein lipase [18] and observed immunogenic responses in final formulations linked to the presence of phospholipase B-like protein in final formulations [21] indicate that HCPs can and do fail to be cleared from mAb products in typical platform processes. Increasing the ability to clear these high-risk species will result in safer biotherapeutic products and more robust processes.

**Figure 1:**
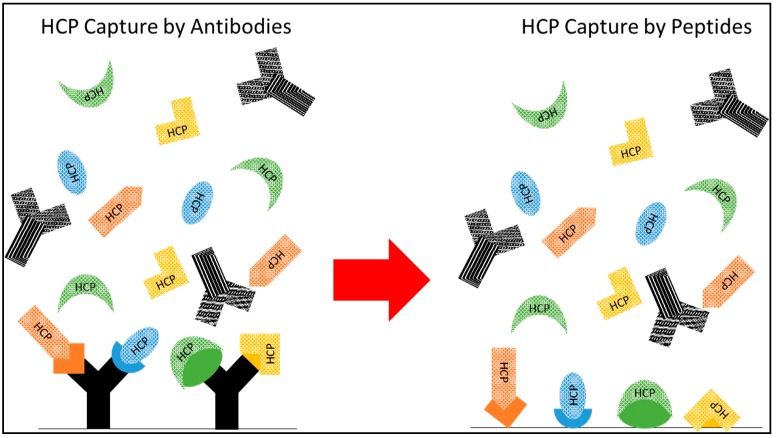
Conceptual diagram of “polyclonal” synthetic HCP-binding resins. Targeted HCP capture is not only possible, but standard practice for HCP quantification using HCP ELISA via polyclonal α-HCP antibodies, as depicted on the left. We propose the generation of a set of diverse ligands to mimic the broad capture of varied HCP species with low binding of IgG, as shown on the right, to allow for targeted capture without the expense and variability introduced by antibody-based ligands.

A promising strategy to achieve these goals is to target these hard-to-remove, problematic HCPs using affinity or pseudo-affinity methods. This paper describes the development of an ensemble of ligands capable of targeted capture of HCPs in flow-through mode to be utilized as next-generation polishing media in mAb manufacturing. While challenging, the specific capture of targets as diverse as HCPs has a precedent in enzyme-linked immunosorbent assays (ELISA) for HCP quantification, wherein HCPs are collectively captured using a mixture of α-HCP polyclonal antibodies [22]. While polyclonal α-HCP antibodies are not economically feasible for the large-scale capture of HCPs, extensive studies have shown that peptide ligands can serve as suitable synthetic alternatives for affinity-like and multimodal binding interactions [23,24,25]. As a result, ensembles of peptide ligands with varied selectivity can simulate the performance of α-HCP polyclonal antibodies (Figure 1) and serve as next-generation multimodal ligands for mAb polishing. Single ligands may either limit overall capture due to a lack of promiscuous binding, or alternatively provide such a low specificity that the product also binds. As a result, it was decided to identify multiple ligands with varied available interaction mechanisms to balance between yield and breadth of HCP capture. Broad capture of diverse HCPs, particularly at low concentrations, with multiple peptide ligands has previously been demonstrated for the enrichment of extremely low concentration proteins for liquid chromatography-tandem mass spectrometry (LC/MS/MS) detection in proteomics applications by Guerrier et al. [26,27]. These results provide confidence that a similar strategy to capture this diverse group of low concentration HCPs using multiple peptide ligands can be successful.

The sections that follow describe the design, construction, and screening of solid phase, combinatorial libraries of linear peptides to identify an ensemble of peptide ligands that bind specifically to Chinese hamster ovary (CHO) HCPs, while showing minimal binding to human IgG. The ligands were selected from a one-bead-one-component (OBOC) peptide library synthesized on HMBA-ChemMatrix (hydroxymethylbenzoic acid-conjugated version of ChemMatrix) resin using a multiplexed fluorescence-based screening method, using CHO HCPs and human IgG labeled respectively with a red (Alexa Fluor 594 or Alexa Fluor 546) and a green (Alexa Fluor 488) fluorescent dye. For primary screening, human polyclonal IgG was chosen over monoclonal antibodies to negatively select against a broad pool of IgGs, ensuring this technology would be applied as a platform for multiple mAb products. The HCPs were generated from a non-producing CHO culture harvest under simulated production conditions. The library beads displaying only red fluorescence (HCP binders) were sorted either manually or by selection using a ClonePix 2 colony picker. The sequencing of the peptides was performed as described in prior work [28,29]. The identified ligands were synthesized on Toyopearl AF-Amino-650M resin, and the resulting adsorbents challenged with clarified harvest from a CHO-K1 mAb production run to evaluate HCP removal in flow-through mode. The values of HCP targeted binding ratio (TBR), defined as the percent of host cell protein captured divided by the percent of IgG lost, for the various peptide adsorbents were compared to reference anion exchange (Capto Q) and multimodal (Capto Adhere) resins. It was found that multipolar peptide ligands have improved HCP targeted binding compared to reference commercial polishing resins, that the performance of hydrophobic/positive resins was similar to that of the tested anion exchange resin Capto Q, and significantly more selective to HCPs than Capto Adhere. These results indicate that resins with peptide ligands may be used for HCP clearance, either in a flow-through mode or weak partitioning mode.

## 2. Results

### 2.1. Library Design, Synthesis, and Screening

**Figure A1:**
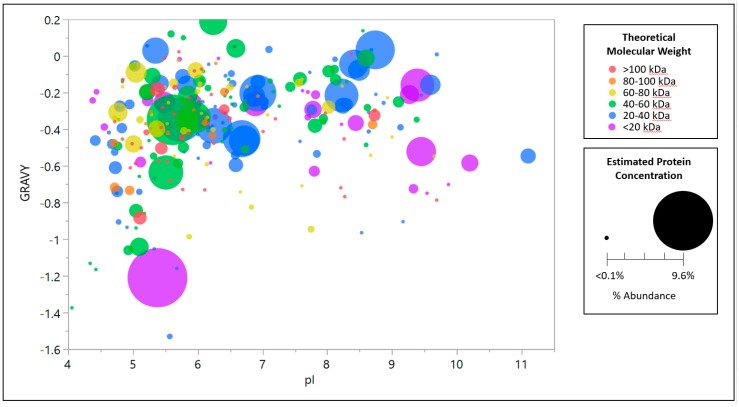
Host cell protein bubble plot distribution by approximate molar percent abundance, theoretical molecular weight, theoretical isoelectric point, and grand average of hydropathy clarified for null CHO-S cell culture harvest material used in this work as the HCP population fluorescently tagged for solid phase peptide library screening for null CHO-S abundance.

The one-bead-one-peptide (OBOP) libraries used for this work were synthesized using the split-couple-recombine method pioneered by Lam et al. [30] to discover synthetic ligands that bind target proteins. Libraries were synthesized on HMBA-ChemMatrix resin, which affords high peptide purity and can be used to probe protein binding. The ChemMatrix resin was selected to allow for fluorescent screening with a strategy adapted from the Camperi research group [31] to minimize false positives as a result of fluorophore–peptide interactions. Given that the majority of HCPs present in the CHO harvest material are hydrophilic and negatively charged under physiological conditions (see Appendix A Figure A1), the amino acid composition was limited to 12 out of the 20 natural amino acids for library construction, namely histidine, arginine, and lysine (positively charged); isoleucine, alanine, and glycine (aliphatic); phenylalanine and/or tyrosine (aromatic), aspartate (negatively charged), serine, and asparagine or glutamine (polar). Notably, narrowing the pool of amino acids reduces the library size and screening time, and aids sequencing. Two libraries were constructed, namely a tetrameric (X_1_-X_2_-X_3_-X_4_-G-S-G) and a hexameric (X_1_-X_2_-X_3_-X_4_-X_5_-X_6_-G-S-G), wherein X_i_ represents a combinatorial position that can be occupied by any of the chosen amino acids, and G-S-G is a glycine-serine-glycine C-terminal spacer. Hexamers have been previously demonstrated as effective small synthetic ligands for pseudo-affinity and low concentration applications by our group and others [23,24,25,32,33]; in addition, shorter tetrapeptides were utilized to determine whether comparable capacity and targeted binding could be obtained at a lower cost-of-goods. Given that the majority of HCPs observed in production harvest is negatively charged, N-terminal amines were left uncapped to allow for ionic interactions with the free amine. The GSG spacer included in the library sequence was used as an inert spacer arm to promote the display of the combinatorial segment, and was used as a tracking sequence in LC/MS/MS peptide sequencing due to frequent occurrence of both the -GSG and -SG y-ion fragments observed. The HMBA linker allows for on-resin deprotection of the side chain functional groups on the amino acid residues prior to library screening; the linker is also alkaline-labile, and enables post-screening cleavage of the peptides from the selected ChemMatrix beads to be finally sequenced using LC/MS/MS. Libraries were screened via incubation with HCPs labeled with Alexa Fluor 594 or Alexa Fluor 546 NHS Esters and IgGs labeled with Alexa Fluor 488 NHS Ester to enable simultaneous screening for ligands that both bind to HCPs and do not bind to IgG. Alexa Fluor fluorescent tags were selected for brightness and high photostability, and to minimize overlap in emission. A volume of ≈5 µL of settled ChemMatrix library resin beads was combined with 10 µL fluorescent protein and incubated overnight at 2–8 °C to ensure saturation of the resin beads. For manual screening, beads were selected by applying the following criteria: (i) IgG maximum fluorescence < 2500 relative fluorescence units (RFU), based on observing the fluorescent intensity range from negative control beads; (ii) HCP maximum fluorescence > 10,000 RFU, to include the upper 50% of beads by HCP max intensity (one-sided upper tolerance interval ≈ 13,500, α = 0.95). Inspection of the fluorescent intensity profile as a function of radius in addition to manual review of the fluorescent images were also implemented for positive beads to ensure the maximum fluorescence signal was not a result of an image artifact or bead defect. For the ClonePix 2 system, bead selection was based on the interior mean intensity parameter developed for the ClonePix system, which is approximately equivalent to the average fluorescent intensity within the bounds of the beads. Beads were selected based on the following gates: (i) fluorescein isothiocyanate (FITC) interior mean intensity < 2500 RFU; (ii) Rhodamine (red) interior mean intensity > 500 RFU, representing a similar ratio of picked beads to the total beads screened (≈20%). While the threshold for bead selection for the HCP fluorescence in this instance may appear substantially lower than observed with the manual screening, differences were expected given that a different Alexa Fluor dye was required for this system (Alexa Fluor 546 [34], which has a lower reported initial brightness compared to Alexa Fluor 594 [35]), in addition to the differences in imaging exposure and intensity required to visualize the beads.

### 2.2. Sequencing of HCP-Binding Ligand Candidates

The selected beads were sequenced following the method developed in prior work [28,29]. First, the isolated beads were copiously rinsed with a 0.2 M acetate buffer (pH 3.7) to remove all bound proteins. Particular care was taken with the beads selected with the ClonePix 2 device to remove the CloneMatrix utilized to immobilize the beads for imaging and picking. The beads were then individually treated with 38 mM sodium hydroxide, 10% *v*/*v* acetonitrile to cleave the ester bond between the GSG spacer and the HMBA linker; to prevent alkaline degradation of the peptide, the exposure to the alkaline solution was limited to 10 min, after which the cleavage solutions was neutralized with an equal volume of 100 mM citrate buffer, pH 3.0, 10% *v*/*v* acetonitrile. The cleaved peptides were then reconstituted in aqueous 0.1% formic acid and sequenced using LC/MS/MS. The peptide sequences were obtained by searching the acquired MS data against the corresponding tetramer and hexamer peptide FASTA databases using MASCOT (Matrix Science). 

The resulting sequences, listed in Table 1, were grouped in three classes based on consensus in amino acid composition, namely (i) hydrophobic/positively charged peptides (HP), which comprise ≈25%–35% of positively-charged residues (R, K, H) and 65%–75% hydrophobic (I, A, F, Y) residues; (ii) multipolar peptides (MP), which comprise one or more positive (R, K, H) and one negative residue (D); and (iii) unclassified residues. Previous work on proteomic identification and quantification of CHO HCPs in both the null harvest and the harvest used herein (see Appendix A Figure A1) has shown that the majority of the HCPs have sequence-based isoelectric points < 7, and are likely negatively charged under physiological conditions. Thus, the persistent identification of peptides featuring positive amino acids is consistent with capture of these species via long-range ionic interactions.

**Figure 2:**
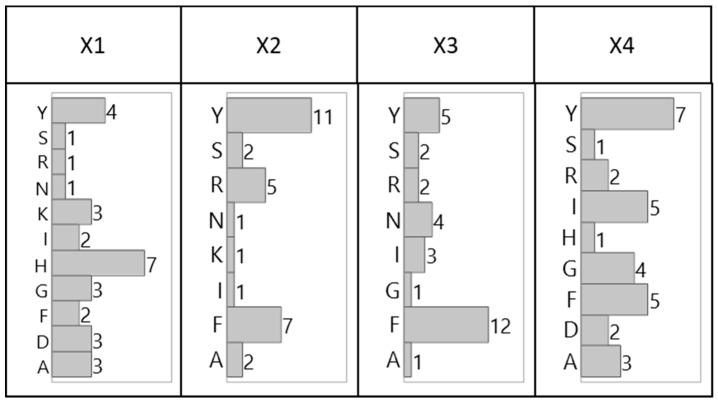
Distribution of amino acid residues for lead tetrameric HCP-binding peptide candidates identified via manually sorted solid-phase fluorescent screening by combinatorial position.

**Figure 3:**
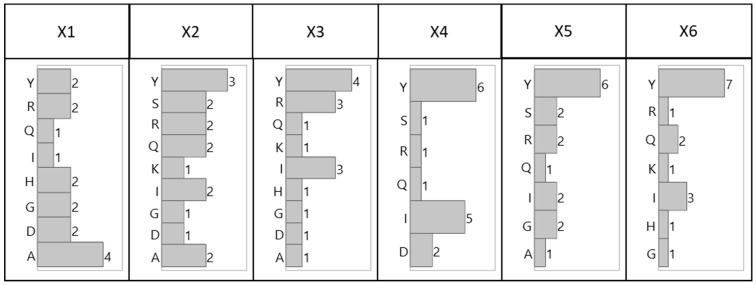
Distribution of amino acid residues for lead hexameric HCP-binding peptide candidates identified by ClonePix 2 sorted solid-phase fluorescent screening by combinatorial position.

The distribution of the amino acids by combinatorial position, shown in Figure 2 (tetrameric) and Figure 3 (hexameric), reveal preferential placement of hydrophobic, particularly aromatic, amino acids towards the C-terminus. This phenomenon, which is especially apparent with hexameric sequences, can be attributed to a sequence-based peptide-HCP affinity across multiple HCP species, or to an unexpected bias in the libraries related to a higher synthetic yield of the observed sequences. The consensus observed within each library and between the two libraries, however, indicates limited bias in either bead selection or sequencing introduced between the two screening methods (manual sorting vs. ClonePix 2 sorting) used for this work.

### 2.3. Secondary Screening of HCP-Binding Ligand Groups by Static Binding Evaluation

**Figure 4:**
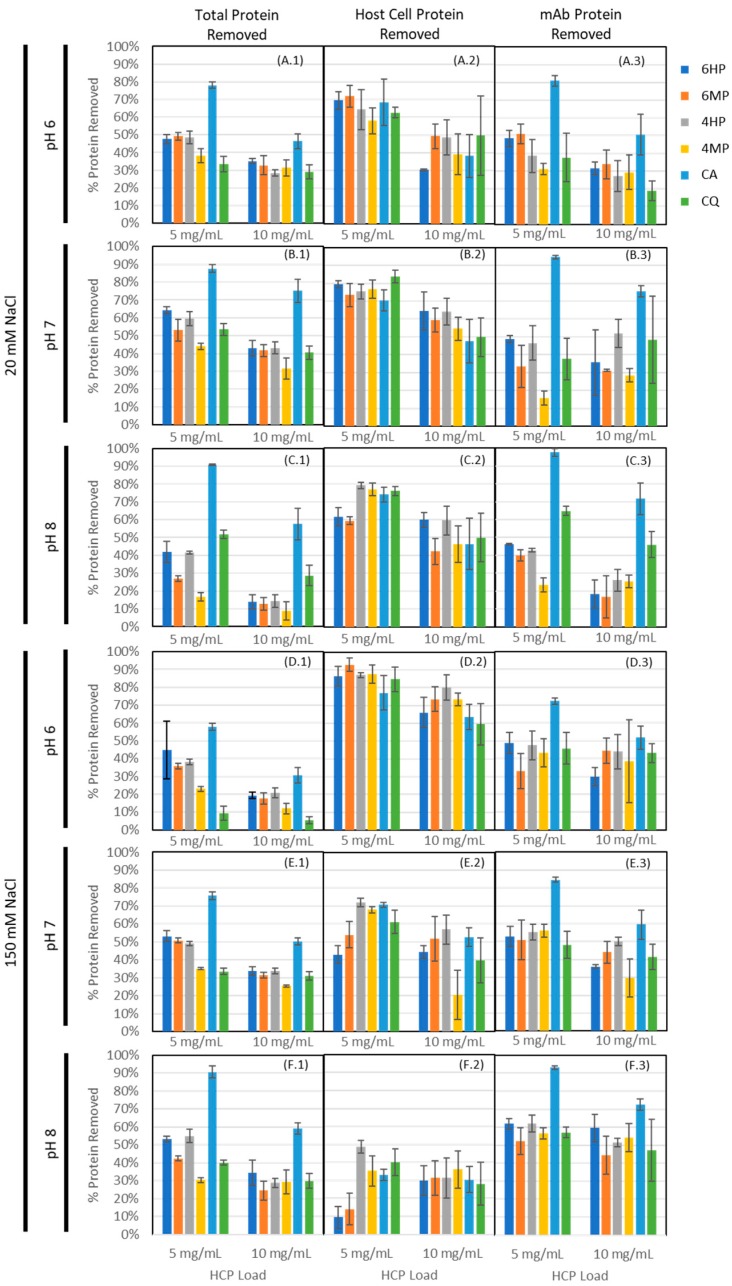
Protein removal (N = 3 for each condition) by hexameric hydrophobic positive and multipolar (6HP and 6MP, respectively) and tetrameric hydrophobic positive and multipolar (4HP and 4MP, respectively) lead HCP-binding peptide ligands coupled to Toyopearl Amino-650M resin in static binding mode, as compared to commercial resins Capto Adhere and Capto Q. Panels A.1–F.1 indicate total protein removal as measured using a Bradford assay. Panels A.2–F.2 indicate CHO-K1 host cell protein removed as measured using a Cygnus CHO HCP ELISA, 3G assay kit. Panels A.3–F.3 indicate monoclonal antibody removed as measured using a Thermo Fisher EasyTiter kit. Each resin was screened in multiple buffer conditions (A panels = pH 6, 20 mM NaCl; B = pH 7, 20 mM NaCl; C = pH 8, 20 mM NaCl; D = pH 6, 150 mM NaCl; E = pH 7, 150 mM NaCl; F = pH 8, 150 mM NaCl); and for two load conditions: ≈5 mg HCP loaded per mL resin, and ≈10 mg HCP loaded per mL resin.

An ensemble of 18 peptides, selected from the groups listed in Table 1, were individually synthesized on Toyopearl AF-Amino-650M resin and mixed into a single heterogeneous adsorbent as follows: (i) 6HP, including sequences GSRYRYGSG, RYYYAIGSG, AAHIYYGSG, IYRIGRGSG, and HSKIYKGSG; (ii) 6MP, including sequences ADRYGHGSG, DRIYYYGSG, DKQRIIGSG, RYYDYGGSG, and YRIDRYGSG; (iii) 4HP, including HYAIGSG, FRYYGSG, HRRYGSG, and RYFFGSG; and (iv) 4MP, including DKSIGSG, DRNIGSG, HYFDGSG, and YRFDGSG. While the initial peptide identification was performed on HMBA-ChemMatrix, this resin was not suitable for subsequent confirmatory studies due to very low binding capacity and resin compressibility. Toyopearl AF-Amino-650M was selected for secondary screening due to its ability to serve as both an efficient resin for solid-phase peptide synthesis and as a rigid, chromatography-compatible resin with reasonable capacity for protein adsorption applications. To ensure that the behavior of these peptide-coupled resins was not a result of the base matrix alone, a preliminary comparison of the peptide-coupled resins to uncoupled Toyopearl AF-Amino-650M and Toyopearl HW-65F (polymethacrylate resin with no amine conjugation) was performed, showing statistically different binding of host cell protein, IgG, and total protein (data available in Appendix C). The adsorbents were evaluated to verify binding capacity and selectivity via equilibrium binding studies at different values of pH (6, 7, and 8) and salt concentration (20 mM and 150 mM) of the binding buffer, using a representative IgG-producing CHO-K1 clarified cell culture harvest; commercial resins Capto Adhere (CA) and Capto Q (CQ) were utilized as controls to establish the threshold for HCP and IgG binding required for flow-through purification steps. Percent protein removal for HCP using HCP ELISA, IgG using Easy-Titer assay, and total protein using Bradford assay are presented in Figure 4 (data tabulated in Appendix B Table A1). 

In evaluating protein capture across the four peptide-based adsorbents, we consistently observed significantly higher binding (*p* < 0.05) of total protein for low salt conditions when compared to high salt conditions at pH 6 and 7 (exceptions were 6MP, pH 7 and 6 HP, pH 6, with *p* = 0.4832 and 0.7832, respectively, indicating no significant difference) for both load conditions, suggesting that, as with Capto Q and Capto Adhere, ionic interactions play a central role in the binding mechanism. The relevance of electrostatic interactions in peptide-HCP binding was anticipated given that the majority of HCPs have theoretical isoelectric points well below neutral pH (pI < 6 ≈46%, pI < 7 ≈66%, pI < 8 ≈71% based on proteomic analysis of the feed stream). Additionally, all species tested in the secondary screening included at least one positively charged amino acid residue and were screened in bis-tris or tris buffer, where the positive buffer ion would interfere minimally with any ionic interactions from positively charged residues. That being said, we observed an increase in total protein binding (*p* = 0.021, 0.077, 0.0012, and 0.0003 for 4HP, 6HP, 4MP, and 6MP, respectively) and IgG binding (*p* = 0.016, 0.010, 0.0005, and 0.088 for 4HP, 6HP, 4MP, and 6MP, respectively) for high salt conditions at pH 8 for peptide resins, where Capto Q maintained significantly higher binding with low salt (*p* = 0.0041 for total protein, *p* = 0.0257 for IgG). This suggests greater influence of other interaction mechanisms outside of strictly ionic interactions from the peptide resins, with the potential for hydrophobic interactions becoming more dominant under these conditions given operation closer to the isoelectric point of the highly abundant IgG.

At the same time, the dependence of total protein (HCP + IgG) binding upon pH varied significantly between Capto Q and the peptide ligands, suggesting that binding on the peptide resins was more sequence-based in nature than for Capto Q. As might be expected, Capto Q and Capto Adhere showed statistically significant changes in binding of IgG as a function of pH (*p* = 0.0296 and 0.0002 for Capto Q and Capto Adhere, respectively) for low salt and low load conditions, where maximum binding occurred at pH 8. In contrast, we observed weaker correlation between pH and binding to the hydrophobic positive resins (*p* = 0.4922 and 0.5353 for 4HP and 6HP, respectively) and statistically significant correlation with maximum binding at pH 6 for the multipolar resins (*p* = 0.0073 and *p* = 0.0819). The differences in mAb binding suggest a distinct binding selectivity of the peptides, under the conditions tested, compared to the Capto Adhere multimodal adsorbent. With both MP and HP resins, we identified binding conditions under which observed HCP removal was comparable to the values given by Capto Q and Capto Adhere resins, while the percentage of mAb loss was equal or lower than that of Capto Q. Moreover, Capto Adhere was found to remove substantially more mAb compared to all other resins, causing a loss of mAb product consistently > 70% across all binding conditions. This indicates that the library screening via multiplexed fluorescence directed peptide selection toward sequences that target HCPs with a degree of affinity higher than the mixed-mode level. Interestingly, HCP capture was more robust for the tetrameric ligands as compared to the hexameric ligands in the higher pH regime (pH 7 and pH 8), where as much as 40% more HCP was captured by the tetrameric ligands than the corresponding hexameric peptides. This effect was arguably the result of higher binding selectivity displayed by peptide ligands with longer sequences, which narrows the interaction range to fewer HCP species.

As expected, a reduced percent removal was observed with increased protein load across all tested adsorbents. This helped to identify the range in which HCP binding is observable under static binding conditions. As both load conditions were incubated for sufficient time to allow binding equilibrium, we screened at a range of load conditions to ensure that the fraction of HCPs captured was measurable in the static binding supernatant. 

### 2.4. Resin Targeted Binding

**Figure 5:**
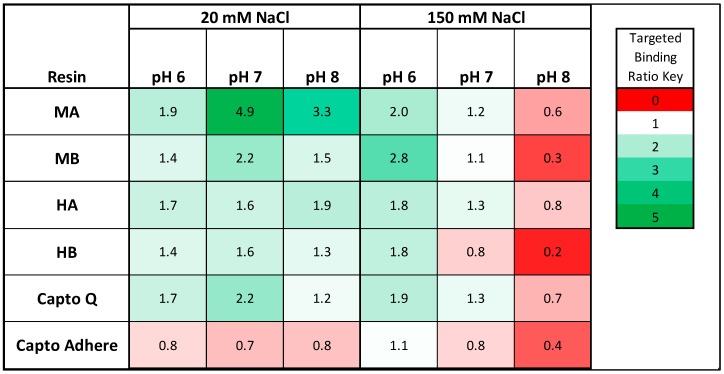
Resin HCP targeted binding ratio (TBR) by resin and buffer condition (N = 3). Resin HCP TBR is defined as the percent of HCP removed compared to the feed stream divided by the percent of mAb removed compared to the feed stream in static binding mode. In this analysis, HCP TBR > 1 indicates preferential binding to HCP as compared to IgG, and HCP TBR < 1 indicates preferential binding to IgG.

The peptide adsorbents were ranked by HCP TBR, herein defined as ratio of host cell protein removed and amount of mAb lost, wherein HCP TBR < 1 indicates preferential binding to mAb, and HCP TBR > 1 indicates preferential binding to CHO HCPs. The values of HCP TBR by resin and buffer condition are summarized for the low load condition (5 mg/mL) in Figure 5. We observed preferential HCP binding by all four peptide adsorbents with most of the binding buffers tested, with the exception of the pH 8, 150 mM NaCl condition. Given that the mAb concentration in the cell culture harvest is at minimum two orders of magnitude higher than any single host cell protein species, as measured in the clarified harvest, the identified peptides exhibited a much stronger binding for HCPs compared to mAb. The preferential binding to IgG observed with peptide resins and Capto Q at the pH 8, under high salt (150 mM) conditions, in addition to the lower HCP TBR observed at pH 7, 150 mM sodium chloride, were likely a result of buffer pH conditions close to or above the isoelectric point of the mAb (measured at ≈7.6 using isoelectric focusing gel) coupled with higher salt concentration, which minimized the contribution of ionic interactions to binding.

Multipolar peptides showed a superior targeted binding for HCPs compared to hydrophobic positive peptides, indicating that they may provide useful alternatives to current mixed-mode ligands for mAb polishing. In particular, the tetrameric 4MP resin offered the highest level of HCP targeted binding of 4.9 at pH 7, 20 mM NaCl, more than double the value afforded by commercial Capto Q (2.2). This result was somewhat unexpected given the lack of multipolar and zwitterionic adsorbents used in the context of biopharmaceutical purification to our knowledge. On the other hand, zwitterionic ligands are utilized in highly specific chromatographic applications, such as enantioselective [36,37] and stereoisomer-selective [37] separations. We hypothesize a mechanism of binding for the multipolar ligands that is quite similar to the double ion pairing mechanisms proposed in enantio- and stereoisomer-selective zwitterionic ligands, wherein strong ionic interaction with the positively charged amino acids on the ligands with negatively charged patches on the target are paired via a weaker ionic interaction with the negatively charged residue with the target in order for the protein target to remain bound. This mechanism could also apply to the hydrophobic/positive ligands and commercial multimodal resins such as Capto Adhere, with the exception that the double-ion pairing interaction mechanism is replaced by other binding mechanisms (π-π bonding, van der Waals interaction, hydrogen bonding, etc.). Should the proposed binding mechanisms proposed be confirmed, the combination of these ligands into a “polyclonal” ensemble would allow for capture of a more diverse set of HCPs than each set alone.

## 3. Discussion

Multipolar and hydrophobic positive peptides show promise as next-generation mixed-mode ligands for CHO HCP capture in IgG purification applications. The 4MP resin in particular was observed to bind approximately a 5-fold higher fraction of soluble HCP impurities compared to mAb from a clarified, diafiltered CHO-K1 cell culture harvest. It was additionally observed that the hexameric multipolar (6MP) peptides and both hexameric and tetrameric hydrophobic positive (6HP and 4HP, respectively) peptides identified preferentially bound host cell protein consistently, particularly for low salt (20 mM NaCl) conditions. Taken together, these novel synthetic peptide ligands may offer interesting alternatives to commercial stationary phases for flow-through mode purification of IgG from a clarified cell culture harvest. This, in turn, could enable more efficient process alternatives, for example, by (i) extending the life of protein A resin due to reduced HCP load in the feed stream, or (ii) enabling replacement of protein A capture steps with a less specific, more cost-effective product capture stationary phase such as cation exchange, multimodal, or Protein A mimetic ligands [23,25,38,39]. This work demonstrates the potential of a combination of peptide ligands to purify IgG under conditions that are close to physiological pH, providing an option for HCP capture prior to product capture steps, allowing more flexibility in process applications.

Future work aims to further characterize these HCP-specific ligands by identifying HCP species that are specifically targeted by each resin group. This will help identify potential opportunities for increasing the ability to capture the full range of HCP species, including the problematic HCPs. Additionally, we will search for gaps where none of the currently identified ligands bind particular problematic HCP species. As the work demonstrated here was performed under static binding conditions, and ligands were screened without optimization of the resin base matrix, linker composition, linker density, or ligand density, future work will focus on optimization of these resin conditions and range finding under dynamic binding conditions. With this further optimization, there is an opportunity for highly selective, scalable HCP capture for the purification of IgG from CHO production harvest. If successful, identifying HCP-specific ligands would significantly lower the manufacturing costs of mAbs by providing a superior technology for HCP capture before the product capture step. Alternatively, polyclonal stationary phases could enable fully flow-through downstream processing by substantially reducing the complexity of the process stream post-HCP capture.

## 4. Materials and Methods

### 4.1. Materials

For synthesis and deprotection, the HMBA-ChemMatrix resin used for library synthesis was obtained from PCAS BioMatrix (Saint-Jean-sur-Richelieu, Canada). Toyopearl AF-Amino-650M resin for secondary screening synthesis, Toyopearl HW-65F, Kaiser test kit, triisopropylsilane (TIPS), and 1,2-ethanedithiol (EDT) were obtained from MilliporeSigma (St. Louis, MO, USA). N′,N′-dimethylformamide (DMF), dichloromethane (DCM), methanol, and N-methyl-2-pyrrolidone (NMP) were obtained from Fisher Chemical (Hampton, NH, USA). Fluorenylmethoxycarbonyl- (Fmoc-) protected amino acids Fmoc-Gly-OH, Fmoc-Ser(But)-OH, Fmoc-Ile-OH, Fmoc-Ala-OH, Fmoc-Phe-OH, Fmoc-Tyr(But)-OH, Fmoc-Asp(OtBu)-OH, Fmoc-His(Trt)-OH, Fmoc-Arg(Pbf)-OH, Fmoc-Lys(Boc)-OH, Fmoc-Asn(Trt)-OH, and Fmoc-Glu(OtBu)-OH in addition to 7-Azabenzotriazol-1-yloxy)tripyrrolidino-phosphonium hexafluorophosphate (HATU), diisopropylethylamine (DIPEA), piperidine, and trifluoroacetic acid (TFA) were obtained from Chem-Impex International (Wood Dale, IL, USA). For peptide sequencing, citric acid, acetonitrile, and formic acid were obtained from Fisher Chemical (St. Louis, MO, USA), ReproSil-Pur 120 C18-AQ, 3-µm resin was obtained from Dr. Maisch GmbH (Ammerbuch-Entringen, Germany), and 25 cm × 100 µm PicoTip or IntegraFrit emmiter columns were obtained from New Objective (Woburn, MA, USA).

The CHO-S cell line, CD CHO AGT™ medium, CD CHO Feed A, glutamine, Pluronic F68, and Anti-Clumping Agent used to generate HCP-containing harvest for fluorescence tagging were provided by Life Technologies (Carlsbad, CA, USA). Antifoam C, sodium phosphate (monobasic), and Tween 20 were obtained from MilliporeSigma (St. Louis, MO, USA). Alexa Fluor 488, 594, and 546 NHS-Activated Esters were obtained from ThermoFisher, and sodium chloride, sodium phosphate (dibasic), sodium hydroxide, hydrochloric acid, bis-tris, and tris were obtained from Fisher Chemical (Hampton, NH, USA). Macrosep Advance 3-kDa MWCO Centrifugal Devices were supplied by Pall Corporation (Ann Arbor, MI, USA), and Amicon Ultra 0.5-mL Centrifugal Filter Unit with 3-kDa MWCO filters were made using EMD Millipore (St. Louis, MO, USA). Lyophilized polyclonal human IgG was obtained from Athens Research (Athens, GA, USA). CloneMatrix for ClonePix 2 screening was generously provided by Molecular Devices (Sunnyvale, CA, USA). The model mAb production CHO-K1 cell culture harvest used for secondary screening was donated by a local biomanufacturing company. Capto Q and Capto Adhere chromatography resins were generously provided by GE Life Sciences (Marlborough, MA, USA). For protein quantification, Pierce Coomassie Plus (Bradford) Assay Kits and Easy-Titer human IgG (H+L) Assay kits were obtained from ThermoFisher (Rockford, IL, USA). CHO HCP ELISA, 3G kits were obtained from Cygnus Technologies (Southport, NC, USA).

### 4.2. Methods

#### 4.2.1. Solid Phase Peptide Synthesis and Deprotection

Solid phase peptide synthesis (SPPS) was used for the generation of both the solid phase peptide libraries and the identified ligands screened for this work. The synthesis and sequencing procedures were performed according to a protocol from Menegatti et al. [29] and adapted for the construction of linear peptides. OBOP libraries for on-bead fluorescence screening were synthesized on ChemMatrix HMBA resin (loading = 0.6 mmol amine/g resin) for the peptide libraries. Lead ligand candidates for chromatographic screening were synthesized on Toyopearl Amino-650M resin (loading = 0.1 mmol amine/mL resin). Synthesis for all resins was performed on a Syro II automated parallel peptide synthesizer (Biotage). Aliquots of 100 mg of ChemMatrix resin or 0.6 mL Toyopearl resin were swelled for 20 min in DMF at 40 °C with intermediate vortexing. Couplings were performed at a 3- to 5-fold molar excess of Fmoc-protected amino acids and HATU, and a 6-fold molar excess of DIPEA solubilized in NMP relative to reactive sites on the resin. The coupling reaction was performed at 45 °C for 20 min with agitation via intermediate vortexing. Each coupling reaction was performed three to four times per cycle prior to Fmoc deprotection to maximize the reaction completion. For deprotection, resins were first washed four times with DMF, then incubated in 20% piperidine for 20 min at room temperature with agitation via intermediate vortexing, followed by an additional wash step as described above. All sequences were synthesized with a C-terminal glycine-serine-glycine (GSG) tail to act as a non-reactive spacer between the peptide sequence and the base matrix. Combinatorial tetrameric (X1-X2-X3-X4-G-S-G) and hexameric (X1-X2-X3-X4-X5-X6-G-S-G) peptide libraries were synthesized as OBOP libraries using the split-couple-recombine method [30]. While coupling efficiency was not directly monitored for each synthesized sequence, this coupling procedure was monitored using a Kaiser test for both Toyopearl AF-Amino-650M and ChemMatrix HMBA for the c-terminal glycine coupling prior to Fmoc deprotection, resulting in negligible observed uncoupled amines on the base resin. For the tetrameric library, combinatorial positions were composed of equal ratios of isoleucine (I), alanine (A), glycine (G), phenylalanine (F), tyrosine (Y), aspartate (D), histidine (H), arginine (R), lysine (K), serine (S), and asparagine (N). The residues selected for the hexameric library were slightly modified by removal of F and N, and inclusion of glutamine (Q) for ease of synthesis and sequencing. Side-chain deprotection for both combinatorial libraries and single-ligand resins was performed by washing resins five times with ≈10 mL DMF, then washing the resins with ≈10 mL DCM and drying the resin with compressed nitrogen until the resin dried to a fine powder (3–5 times). A cocktail of 94% TFA, 1% EDT, 3% TIPS, and 2% deionized water was then incubated with the resin (6 mL deprotection cocktail per 100 mg resin) on a rotator at room temperature for 2 h. Resins were washed three to five times first with DMF then 20% methanol and stored in 20% methanol at 2–8 °C.

#### 4.2.2. CHO-S Culture and Harvest for Host Cell Protein Production

CHO cell lines were selected as our model system to obtain typical HCP profiles found in biotherapeutics processes. CHO-S cell culture harvest was provided by the Biomanufacturing Training and Education Center (BTEC) at North Carolina State University and was cultured according to their standard procedure for expansion and production of the CHO-S wild-type (WT) cell line. Briefly, the CHO cell culture bulk fluid (CCBF) was from a null CHO-S cell line grown in CD CHO AGT™ medium with 4 mM glutamine and 1 g/L pluronic F68. The cultures were fed 5% daily with CD CHO Feed A from days 3–10. The cultures were also supplemented with 0.1% Anti-Clumping Agent to prevent cell aggregation. Antifoam C was added at 10 ppm to prevent foaming in the bioreactor. CD CHO AGT™ medium contains no proteins or peptide components of animal, plant, or synthetic origin, as well as no undefined lysates or hydrolysates. The cell culture process was operated at a set pH of 7.0 ± 0.30, 37.0 °C, and 50.0% dissolved oxygen concentration. Post-production, the CHO-S harvest was clarified via centrifugation at 8000× *g* for 30 min. The supernatant was then filtered with a 0.2 µm polyethersulfone (PES) membrane using VWR Full Assembly Bottle-Top vacuum filters.

#### 4.2.3. Fluorescent Labeling of IgG and CHO-S HCPs

HCPs and IgG were fluorescently labeled with Alexa Fluor NHS esters as guided by the manufacturer’s recommendations [31]. Briefly, wild-type CHO-S clarified harvest was concentrated to 2.3 g protein/L (≈6-fold) and diafiltered into 50 mM sodium phosphate, 20 mM sodium chloride, pH 8.3 using Macrosep Advance 3-kDa MWCO Centrifugal Devices. Lyophilized polyclonal human IgG (Athens Research) was dissolved in 50 mM sodium phosphate, 20 mM NaCl, pH 8.3 at a concentration of 5 g/L. 1 mg Alexa Fluor 596 NHS Ester (AF596) or Alexa Fluor 546 NHS Ester (AF546) for the HCP solution (based on the instrument to be used for fluorescence screening) and 1 mg Alexa Fluor 488 NHS Ester (AF488) for the IgG solution were each dissolved in 100 µL extra dry DMF, which was immediately combined with 1 mL of the diafiltered harvest (HCP-AF596 or HCP-AF546) or IgG (IgG-AF488) and incubated at room temperature on a rotator for 1 h. After incubation, the samples were diafiltered into 50 mM sodium phosphate, 150 mM sodium chloride, pH 7.4 using Amicon Ultra 0.5-mL Centrifugal Filter Unit with 3-kDa MWCO filters to remove unreacted Alexa Fluor dye.

#### 4.2.4. Fluorescence Screening of Solid Phase Peptide Libraries Against IgG and CHO-S HCPs

The hexameric or tetrameric deprotected libraries were washed three times in 50 mM sodium phosphate, 150 mM sodium chloride, pH 7.4 (PBS) at 5× the settled resin volume to equilibrate. HCP-AF596 or HCP-AF546 and IgG-AF488 were diluted in 50 mM sodium phosphate, 150 mM sodium chloride, 0.2% Tween, pH 7.4 for a final concentration of ≈1.3 mg/mL IgG-AF488, ≈0.58 mg/mL HCP-AF546 or HCP-AF596, 50 mM sodium phosphate, 150 mM sodium chloride, 0.1% Tween 20, and mixed with the washed, equilibrated libraries and incubated at 2–8°C overnight. After incubation, the excess protein solution was removed and the resin beads were washed with 50 mM sodium phosphate, 150 mM sodium chloride, 0.1% Tween 20, pH 7.4 (PBS-T). For manual fluorescence screening, the resin was deposited 1 bead per well in a 96-well plate in 40 µL PBS-T, then imaged at 10× magnification using fluorescence microscopy using a Leica DMi8 inverted microscope with a Hamamatsu C13440 digital camera and equipped with a Lumencor spectra light engine in the San-Miguel Lab at NC State University. Lead candidate beads were selected based on the highest observed emission intensity at 630 nm with excitation at 560 nm for Alexa Fluor 594 fluorescence measurement after thresholding based on 510 nm emission intensity at 480 nm excitation for Alexa Fluor 488 fluorescence measurement.

To increase throughput, a ClonePix 2 colony picker was used for fluorescent imaging and higher throughput sorting of HCP positive and IgG negative beads in collaboration with Molecular Devices in Sunnyvale, CA, USA. The colony picker was identified as a possible option to increase throughput due to (1) its ability to quickly image and quantify intensity of large quantities of beads, and (2) the size range of the ChemMatrix beads, which are similar to colonies traditionally picked using the ClonePix 2 instrument. After library incubation with fluorescently tagged proteins and washed as described above, they were suspended in a semi-solid matrix to accommodate imaging and picking. The semi-solid matrix was prepared from two parts Molecular Devices CloneMatrix and three parts 83.3 mM sodium phosphate, 250 mM NaCl, 0.17% Tween 20 to generate a matrix with buffer conditions similar to the protein binding condition used. Approximately 5 to 10 µL settled volume of incubated library was gently incorporated into the matrix solution, then evenly aliquoted across a 6-well plate to obtain a target bead density of ≈100–200 beads per well. The plates were then incubated at 37 °C for 2–18 h to cure the matrix. Plates were imaged using the ClonePix FITC (800 ms exposure, 128 LED intensity) and Rhod (500 ms, 128 LED intensity) laser lines to monitor the presence of Alexa Fluor 488 and Alexa Fluor 546, respectively. Due to slight autofluorescence of the ChemMatrix beads under the FITC filter, bead location (i.e., ClonePix 2 run “Prime Configuration”) was assigned based on fluorescence intensity from the FITC filter. Beads were picked for further processing based on the following characteristics using the ClonePix 2: FITC interior mean intensity < 2500, Rhod interior mean intensity > 100, and 0.05–0.25 mm radius. Picking was performed in suspension mode, with 20 µL aspiration volume to pick up the bead, and a 60 µL expel volume, where excess volume above the aspirated liquid was water.

#### 4.2.5. Lead Peptide Sequencing by LC/MS/MS

Beads selected based on fluorescence were sequenced using an LC/MS/MS approach to determine lead peptide candidates for HCP-binding. Cleavage was performed as described by Kish et al. [28]. Briefly, beads that were positive for HCP fluorescence and negative for IgG fluorescence were first treated with 20 µL 0.2 M acetate, pH 3.7, for 1 h to elute bound protein. Beads were then washed three times with deionized water, then incubated with 10 µL 38 mM sodium hydroxide, 10% *v*/*v* acetonitrile to cleave the peptide from the resin. The cleavage solution was then neutralized with 100 mM citrate buffer, 10% *v*/*v* acetonitrile, then filtered through a fritted pipette tip to remove particulate before drying the resulting solute by speed-vacuum. The powder was then resuspended in 0.1% formic acid for injection onto LC/MS/MS.

A Waters Q-ToF Premier equipped with a nanoAcquity UPLC system with a nanoflow ESI source in the Department of Molecular and Structural Biochemistry at NC State University was used for manually screened, tetrameric candidates, while a Thermo Orbitrap Elite with a Thermo EASY-nLC 1000 was used for hexameric peptide sequences from ClonePix2 screening. Chromatographic separation of the peptide samples was performed with a with a 25 cm × 100 µm PicoTip or IntegraFrit emmiter column packed with ReproSil-Pur 120 C18-AQ, 3-µm resin. Samples were loaded as 10–15 µL injections and separated by a 30-min linear gradient at 300 nL/min of mobile phase A (0.1% formic acid) and mobile phase B (0.1% formic acid in acetonitrile) from 5–40% mobile phase B.

For samples sequenced by the Orbitrap Elite, MS/MS sequencing was performed as follows: positive ion mode, acquisition—full scan (*m*/*z* 350–1250), 60,000 resolving power, MS/MS using a top 5 data dependent acquisition mode with two fragmentation events at settings of 27 and 35 normalized collision energy (NCE) for higher-energy collisional dissociation (HCD) acquisition for each interrogated precursor. Raw LC/MS/MS data were processed using Proteome Discoverer 1.4.1.14 provided through the Department of Molecular and Structural Biochemistry at NC State University. Database searching was performed using MASCOT with a 50-ppm precursor mass tolerance and 50-ppm fragment tolerance against a FASTA formatted database of all possible peptide species in the combinatorial library. Specified modifications included a dynamic modification of each amino acid residue that included a side-chain protecting group during synthesis to account for incomplete side-chain deprotection of the library.

For samples sequenced using Waters Q-ToF Premier, MS/MS sequencing was performed as follows: positive ion mode, acquisition—full scan (*m*/*z* 400–1990), MS/MS using a top 8 acquisition with data dependent acquisition disabled. The default collision energy setting for the instrument based on charge state recognition was used. Raw LC/MS/MS data was processed using ProteinLynx Global Server 2.4. Searching was performed using MASCOT with a 50-ppm precursor mass tolerance and 50-ppm fragment tolerance against a FASTA formatted database of all possible peptide species in the combinatorial library. In cases where more than one peptide match was found for a particular bead, peptides were assigned based on the lowest expectation value. Cases where this occurred generally consisted of multiple peptides identified with identical composition, but a different order of amino acid residues, which was likely a result of the difficulty in distinguishing flipped combinatorial positions in a degenerate library, particularly in cases where there was a low probability of fragmentation at particular positions.

#### 4.2.6. Secondary Screening Static Binding Studies

For secondary screening, a mAb production clarified cell culture harvest derived from a CHO-K1 wild-type cell line was graciously provided by Fujifilm Diosynth (RTP, NC) for use as feed material. Clarified cell culture harvest was concentrated by a factor of ≈4× (≈1.2 mg/mL host cell protein) to model the expected HCP profile after initial concentration using single-pass tangential flow filtration (SPTFF). This was done using Macrosep Advance 3-kDa MWCO Centrifugal Devices. Concentrated harvest was then diafiltered into the appropriate Bis-Tris or Tris buffer as per load condition. For pH 6 and 7 conditions, 10 mM Bis-Tris buffer solutions were used, and 10 mM Tris was used for pH 8 conditions, with “low” and “high” salt buffers composed of 20 mM NaCl and 150 mM NaCl, respectively. Lead candidate Toyopearl resins (6HP, 6MP, 4HP, 4MP) were tested alongside commercially available resins common in flow-through polishing steps for mammalian IgG production, Capto Q, and Capto Adhere. Resins were aliquoted into 1-mL solid-phase extraction (SPE) tubes at 25 µL settled resin volume and washed with 3 × 500 µL of the appropriate load buffer. Resins were then incubated with the diafiltered CHO-S harvest for 1 h on a rotator at HCP loads of ≈5 and 10 mg HCP/mL resin and the resulting supernatant was collected. The resins were then washed with 500 µL load buffer, and the wash and flow-through samples were pooled for analysis.

#### 4.2.7. Quantification of Total Protein, Host Cell Protein, and IgG Removal

Total protein concentrations for samples pre- and post-treatment were measured using a Bradford assay with a Pierce Coomassie Plus (Bradford) Assay Kit (Thermo Fisher, Rockford, IL, USA). IgG concentration for the monoclonal IgG was determined using Thermo Scientific Easy-Titer Human IgG (H+L) Assay Kit. Relative CHO HCP abundance was monitored using a Cygnus CHO HCP ELISA Kit, 3G. Absolute values for HCP concentration were not determined using this assay due to the use of a manufacturer-provided reference standard that did not account for the specific cell line or buffer condition used. To approximate HCP concentration, a correction factor was used per buffer condition to scale the observed concentrations based on the known HCP content in the feed stream. Percent removal for HCP, IgG, and total protein was calculated as described in Equation (1):

(1) $$\mathrm{Percent}\;\mathrm{Removal}\;=\;\frac{V_{Load}\times C_{Load}-V_{FT+Wash}\times C_{FT+Wash}}{V_{Load}\times C_{Load}}\times 100\%$$

C: protein concentration in mg/mL
V: Volume from the relevant fraction in mL

Resin HCP TBR, a metric to describe relative selectivity of the stationary phases developed towards HCP, was calculated as described in Equation (2).

(2) $$Resin\;HCP\;TBR\;=\;\frac{\%\;HCP\;Removed}{\%\;mAb\;Removed}$$

## 5. Patents

Menegatti, Stefano; Lavoie, R. Ashton; di Fazio, Alice; Carbonell, Ruben G. Peptide Ligands for Capture of Host Cell Proteins. U.S. Provisional Patent Application No. 62/784,104, 21 December 2018.

## Figures and Tables

**Table 1:** Lead HCP-binding peptide candidates. The sequences specified here were determined via comparison of LC/MS/MS spectra to a FASTA sequence library of all possible peptide sequences in the combinatorial library from the combinatorial library beads that were identified as HCP-positive and IgG-negative solid phase fluorescent screening studies.

Library	Positive/Hydrophobic	Multipolar	Unclassified
**Hexameric**	AAHIYY-GSG GSRYRY-GSG HSKIYK-GSG IYRIGR-GSG RYYYAI-GSG	ADRYGH-GSG DKQRII-GSG DRIYYY-GSG RYYDYG-GSG YRIDRY-GSG	AAIIYY-GSG GIDQYY-GSG HQASSQ-GSG QQYIII-GSG
**Tetrameric**	AFNA-GSG KFFF-GSG AFYH-GSG KYGY-GSG FRYY-GSG KYFF-GSG HFFA-GSG HFIF-GSG RYFF-GSG HNFI-GSG YRFF-GSG YYFR-GSG HYAI-GSG HYFR-GSG HRRY-GSG	DKSI-GSG DRNI-GSG HYFD-GSG YRFD-GSG	AIYF-GSG NYRS-GSG DFNY-GSG GSIG-GSG GSSY-GSG GFYG-GSG IAFG-GSG IYYA-GSG SYIY-GSG YAFG-GSG

**Figure A2:**
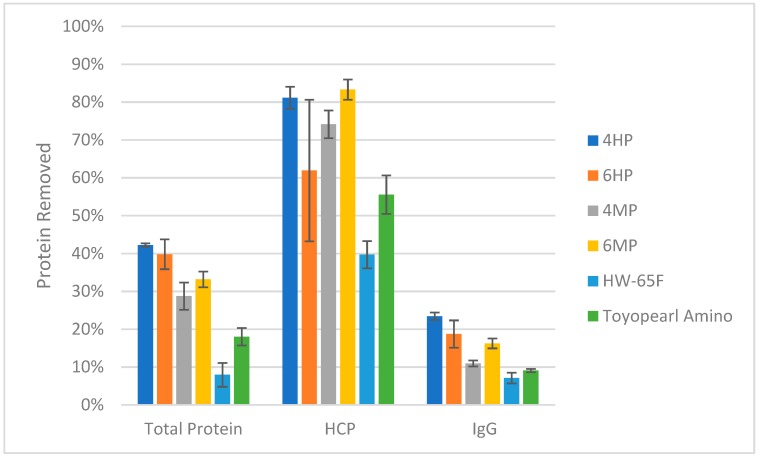
Head-to-head comparison of peptide-coupled resins to Toyopearl AF-Amino-650M and Toyopearl HW-65F polymethacrylate control resins. A representative clarified CHO-K1 mAb production harvest was directly loaded onto the resins at a load of ≈5 mg HCP/ mL resin, where HCP, IgG, and total protein removal in the flow-through were further compared to determine the impact of the peptide coupling. Using an analysis of variance for each assay as a function of resin type, a strong correlation between protein binding and the functional group for each of the resins was found (*p* < 0.0001, =0.0002, <0.0001 for total protein removal, HCP removal, and IgG removal, respectively).

## Abbreviations

HCP	Host cell protein
CHO	Chinese hamster ovary
SPARC	Secreted protein acidic and cysteine-rich
FT	Flow-through
HMBA	Hydroxymethylbenzoic acid
SPPS	Solid phase peptide synthesis
LC/MS/MS	Liquid chromatography-tandem mass spectrometry
OBOP	One-bead-one-peptide
DMF	N′,N′-dimethylformamide
Fmoc	fluorenylmethyloxycarbonyl
HATU	7-Azabenzotriazol-1-yloxy)tripyrrolidino-phosphonium hexafluorophosphate
DIPEA	diisopropylethylamine
NMP	N-methyl-2-pyrrolidone
TFA	Trifluoroacetic acid
EDT	Ethanedithiol
TIPS	triisopropylsilane
mAb	Monoclonal antibody
IgG	Immunoglobulin
PES	Polyethersulfone
Q-ToF	Quadrupole time-of-flight mass spectrometer
UPLC	Ultra performance liquid chromatography
ESI	Electrospray ionization

## Appendix A

Species of CHO HCP are tabulated by abundance as calculated by intensity-based absolute quantification (iBAQ) as determined by proteomic identification and quantification of the null CHO-S clarified harvest material used for fluorescent screening of the solid phase combinatorial peptide library. Concentrated, diafiltered CHO-S harvest and supernatant samples were prepared for proteomic analysis via filter-aided sample preparation (FASP) with a modified trypsin digest adapted from the method described by Wiśniewski and coworkers [40]. For LC/MS/MS analysis, an EASY-nLC 1000 UPLC coupled to an Orbitrap Elite mass spectrometer (Thermo Scientific, San Jose, CA, USA) was used. Chromatographic separation of the FASP digested samples was performed with a 25 cm × 100 µm PicoTip column (New Objective, Woburn, MA, USA) packed with ReproSil-Pur 120 C18-AQ, 3 µm resin (Dr. Maisch GmbH, Ammerbuch-Entringen, Germany). Samples were loaded as 15-µL injections and proteins were separated using a 120 min linear gradient at 300 nL/min of mobile phase A (0.1% formic acid in 2% acetonitrile) and mobile phase B (0.1% formic acid in acetonitrile) from 5–40% mobile phase B. The orbitrap was operated as follows: positive ion mode, acquisition—full scan (*m*/*z* 400–2000) with 60,000 resolving power, MS/MS acquisition using a top 5 data dependent acquisition implementing higher-energy collisional dissociation (HCD) with a normalized collision energy (NCE) setting of 35%. Dynamic exclusion was utilized to maximize the depth of proteome coverage by minimizing re-interrogation of previously sampled precursor ions. Real-time lock mass correction using the polydimethylcyclosiloxane ion at *m*/*z* 445.120025 was utilized to minimize precursor and product ion mass measurement errors [41]. Raw LC/MS/MS data were processed using Proteome Discoverer 1.4 (Thermo Fisher, San Jose, CA, USA). Searching was performed with a 10-ppm precursor mass tolerance and 0.01-Da fragment tolerance with the *Cricetulus griseus* subset of the UniProtKB/Swiss-Prot database with added sequence data for bovine serum albumin (acquisition ID P02769). The database search settings were specific for trypsin digestion with a maximum of one missed cleavage. Specified modifications included dynamic Met oxidation and static Cys carbamidomethylation. Identifications were filtered to a strict protein false discovery rate (FDR) of 1% and relaxed FDR of 5% using the Percolator node in Proteome Discoverer. Based on the sequence of each identified protein, the theoretical isoelectric point (pI) and grand average of hydropathy (GRAVY) were calculated as a model for empirical isoelectric point and hydrophobicity respectively, in addition to the calculation of molecular weight (MW). GRAVY is a metric for hydrophobicity determined as the sum of the contributions of each amino acid in the protein sequence based on the water–vapor transfer free energies and interior–exterior distribution of amino acid side chains [42]. A negative GRAVY value indicates hydrophilic character whereas a positive value indicates hydrophobicity. GRAVY values were calculated using the GRAVY Calculator [43] developed by Stephan Fuchs at University of Greifswald. Theoretical pI and MW were calculated using the ExPASy Bioinformatics Resource Portal Compute pI/Mw tool [44].

## Appendix B

**Table A1:** Tabulated protein removal by resin and buffer condition. Total protein (TP), host cell protein (HCP), and monoclonal antibody (mAb) mass was calculated from the Bradford total protein determination, HCP ELISA, and EasyTiter Assay (see Materials and Methods section), multiplied by the total volume loaded for the load condition, and cumulative flow-through and wash volume collected post-static binding study. Total protein, host cell protein, and mAb removed was calculated as described in Equation (1). The resin HCP targeted binding ratio (TBR) was calculated as described in Equation (2).

Sample	Output	Low Load (≈5 mg HCP/mL Resin)						High Load (≈10 mg HCP/mL Resin)					
		20 mM NaCl			150 mM NaCl			20 mM NaCl			150 mM NaCl		
		pH 6	pH 7	pH 8	pH 6	pH 7	pH 8	pH 6	pH 7	pH 8	pH 6	pH 7	pH 8
Load	TP Mass (mg)	1.3 ± 0.038	1.1 ± 0.038	1.0 ± 0.015	1.1 ± 0.032	1.3 ± 0.027	0.9 ± 0.023	2.6 ± 0.048	2.2 ± 0.034	2.0 ± 0.048	2.1 ± 0.012	2.6 ± 0.041	1.8 ± 0.065
	HCP Mass (mg)	0.14 ± 0.0042	0.12 ± 0.0042	0.11 ± 0.0016	0.073 ± 0.0022	0.15 ± 0.0029	0.099 ± 0.0025	0.28 ± 0.0053	0.25 ± 0.0039	0.22 ± 0.0053	0.15 ± 0.00084	0.28 ± 0.0046	0.20 ± 0.0072
	mAb Mass (mg)	1.1 ± 0.034	0.91 ± 0.032	1.1 ± 0.015	1.3 ± 0.038	1.6 ± 0.031	1.1 ± 0.028	2.3 ± 0.042	0.8 ± 0.300	2.1 ± 0.050	2.5 ± 0.015	3.0 ± 0.048	2.2 ± 0.080
6HP	TP Mass (mg)	0.65 ± 0.020	0.42 ± 0.021	0.60 ± 0.074	0.58 ± 0.19	0.63 ± 0.042	0.42 ± 0.028	1.7 ± 0.021	1.3 ± 0.077	1.8 ± 0.11	1.7 ± 0.030	1.7 ± 0.057	1.2 ± 0.17
	TP Removed (%)	48% ± 2.6%	62% ± 1.9%	42% ± 5.9%	45% ± 16%	53% ± 2.9%	53% ± 1.4%	35% ± 1.3%	43% ± 4.4%	14% ± 3.9%	19% ± 1.8%	34% ± 2.5%	35% ± 7.1%
	HCP Mass (mg)	0.042 ± 0.0061	0.025 ± 0.0024	0.044 ± 0.0047	0.016 ± 0.0068	0.084 ± 0.0079	0.090 ± 0.0039	0.20 ± 0.0073	0.089 ± 0.027	0.090 ± 0.0078	0.078 ± 0.019	0.160 ± 0.0090	0.14 ± 0.021
	HCP Removed (%)	70% ± 4.8%	79% ± 2.0%	62% ± 5.1%	86% ± 5.5%	43% ± 5.0%	9.4% ± 5.9%	30% ± 0.53%	64% ± 11%	60% ± 4.1%	66% ± 8.5%	44% ± 3.6%	30% ± 8.2%
	mAb Mass (mg)	0.57 ± 0.056	0.48 ± 0.018	0.58 ± 0.010	0.63 ± 0.078	0.74 ± 0.091	0.42 ± 0.040	1.6 ± 0.035	0.99 ± 0.28	1.7 ± 0.18	1.74 ± 0.089	2.0 ± 0.15	1.0 ± 0.26
	mAb Removed (%)	48% ± 4.4%	49% ± 1.9%	46% ± 0.5%	49% ± 6.0%	53% ± 5.5%	62% ± 3.0%	30% ± 3.6%	36% ± 18%	21% ± 7.2%	31% ± 3.8%	34% ± 4.1%	54% ± 11%
	HCP TBR	1.4 ± 0.11	1.6 ± 0.03	1.3 ± 0.10	1.8 ± 0.20	0.8 ± 0.03	0.2 ± 0.09	1.0 ± 0.13	2.0 ± 0.73	3.2 ± 1.6	2.2 ± 0.045	1.3 ± 0.29	0.55 ± 0.11
6MP	TP Mass (mg)	0.66 ± 0.031	0.58 ± 0.068	0.75 ± 0.016	0.68 ± 0.025	0.66 ± 0.021	0.53 ± 0.016	1.7 ± 0.18	1.3 ± 0.054	1.8 ± 0.026	1.7 ± 0.056	1.8 ± 0.054	1.3 ± 0.087
	TP Removed (%)	49% ± 2.2%	48% ± 6.1%	27% ± 1.5%	36% ± 1.6%	51% ± 1.4%	42% ± 1.3%	33% ± 5.3%	42% ± 3.2%	13% ± 3.5%	18% ± 3.0%	31% ± 1.6%	24% ± 5.2%
	HCP Mass (mg)	0.040 ± 0.010	0.033 ± 0.0083	0.046 ± 0.0034	0.0084 ± 0.0046	0.068 ± 0.011	0.086 ± 0.0093	0.14 ± 0.016	0.099 ± 0.015	0.13 ± 0.016	0.061 ± 0.016	0.14 ± 0.034	0.13 ± 0.018
	HCP Removed (%)	72% ± 6.0%	73% ± 6.5%	59% ± 2.3%	93% ± 3.8%	54% ± 7.3%	14% ± 8.8%	49.4% ± 6.9%	59.3% ± 6.7%	42.2% ± 7.4%	73.5% ± 6.9%	51.7% ± 12.4%	31.3% ± 9.6%
	mAb Mass (mg)	0.565 ± 0.054	0.620 ± 0.110	0.642 ± 0.036	0.853 ± 0.128	0.770 ± 0.169	0.530 ± 0.087	1.5 ± 0.19	1.0 ± 0.022	1.7 ± 0.24	1.4 ± 0.17	1.7 ± 0.20	1.2 ± 0.22
	mAb Removed (%)	51% ± 5.4%	33% ± 12%	40% ± 3.3%	33% ± 10%	51% ± 11%	52% ± 7.5%	34% ± 8.2%	31% ± 0.56%	17% ± 12%	44% ± 6.9%	44% ± 6.2%	44% ± 10%
	HCP TBR	1.4 ± 0.26	2.4 ± 1.1	1.5 ± 0.086	3.0 ± 0.74	1.1 ± 0.37	0.27 ± 0.17	1.5 ± 0.36	1.9 ± 0.25	5.3 ± 6.1	1.7 ± 0.12	1.2 ± 0.37	0.70 ± 0.063
4HP	TP Mass (mg)	0.67 ± 0.050	0.43 ± 0.011	0.61 ± 0.010	0.66 ± 0.017	0.66 ± 0.043	0.40 ± 0.033	1.8 ± 0.035	1.2 ± 0.046	1.8 ± 0.103	1.7 ± 0.057	1.7 ± 0.067	1.2 ± 0.071
	TP Removed (%)	48% ± 3.6%	58% ± 3.9%	42% ± 0.8%	38% ± 1.5%	49% ± 1.3%	55% ± 3.6%	28% ± 1.7%	43% ± 3.4%	14% ± 3.7%	21% ± 2.7%	34% ± 1.6%	29% ± 2.7%
	HCP Mass (mg)	0.051 ± 0.016	0.028 ± 0.0065	0.024 ± 0.0021	0.015 ± 0.0015	0.040 ± 0.0046	0.051 ± 0.0034	0.14 ± 0.026	0.088 ± 0.016	0.092 ± 0.019	0.046 ± 0.016	0.12 ± 0.022	0.13 ± 0.018
	HCP Removed (%)	64% ± 11%	75% ± 4.2%	79% ± 1.8%	87% ± 1.3%	72% ± 2.3%	49% ± 3.4%	49% ± 9.7%	64% ± 7.4%	59% ± 8.2%	80% ± 7.1%	57% ± 7.9%	31% ± 11%
	mAb Mass (mg)	0.71 ± 0.11	0.46 ± 0.050	0.62 ± 0.010	0.67 ± 0.11	0.68 ± 0.091	0.41 ± 0.045	1.6 ± 0.21	0.7 ± 0.12	1.6 ± 0.14	1.4 ± 0.24	1.5 ± 0.090	1.0 ± 0.088
	mAb Removed (%)	39% ± 9.4%	47% ± 9.5%	43% ± 1.0%	47% ± 8.1%	55% ± 4.3%	62% ± 4.7%	27% ± 8.9%	52% ± 8.0%	26% ± 6.2%	44% ± 9.5%	50% ± 2.4%	51% ± 2.4%
	HCP TBR	1.7 ± 0.30	1.6 ± 0.2	1.9 ± 0.032	1.8 ± 0.17	1.3 ± 0.084	0.78 ± 0.10	1.8 ± 0.38	1.2 ± 0.19	2.3 ± 0.27	1.8 ± 0.23	1.1 ± 0.15	0.61 ± 0.36
4MP	TP Mass (mg)	0.82 ± 0.073	0.64 ± 0.017	0.88 ± 0.025	0.83 ± 0.020	0.87 ± 0.006	0.62 ± 0.013	1.8 ± 0.15	1.5 ± 0.15	1.9 ± 0.14	1.8 ± 0.065	1.9 ± 0.03	1.3 ± 0.13
	TP Removed (%)	38% ± 3.9%	42% ± 1.7%	17% ± 2.2%	23% ± 1.4%	35% ± 0.6%	30% ± 1.5%	31% ± 4.5%	32% ± 6.0%	9% ± 5.3%	12% ± 3.0%	25% ± 0.6%	29% ± 6.7%
	HCP Mass (mg)	0.061 ± 0.011	0.028 ± 0.0061	0.027 ± 0.0042	0.014 ± 0.0059	0.047 ± 0.0032	0.064 ± 0.0070	0.17 ± 0.035	0.11 ± 0.017	0.12 ± 0.023	0.061 ± 0.0081	0.23 ± 0.041	0.13 ± 0.021
	HCP Removed (%)	58% ± 7.3%	77% ± 5.1%	77% ± 3.6%	88% ± 5.1%	68% ± 1.8%	35% ± 8.4%	39% ± 11%	55% ± 6.5%	46% ± 10%	73% ± 3.5%	20% ± 14%	36% ± 10%
	mAb Mass (mg)	0.80 ± 0.017	0.77 ± 0.039	0.84 ± 0.047	0.73 ± 0.095	0.69 ± 0.059	0.47 ± 0.024	1.6 ± 0.23	1.1 ± 0.046	1.6 ± 0.080	1.5 ± 0.58	2.1 ± 0.28	1.0 ± 0.17
	mAb Removed (%)	31% ± 3.1%	16% ± 4.1%	23% ± 4.0%	43% ± 7.8%	56% ± 3.6%	57% ± 3.0%	29% ± 9.6%	28% ± 3.9%	25% ± 3.6%	39% ± 23.4%	30% ± 10.7%	54% ± 7.8%
	HCP TBR	1.9 ± 0.16	4.9 ± 0.27	3.3 ± 0.18	2.0 ± 0.19	1.2 ± 0.069	0.63 ± 0.24	1.3 ± 0.44	1.9 ± 0.18	1.8 ± 0.27	1.9 ± 0.61	0.68 ± 0.77	0.66 ± 0.32
CA	TP Mass (mg)	0.29 ± 0.032	0.11 ± 0.030	0.095 ± 0.0036	0.45 ± 0.016	0.32 ± 0.028	0.083 ± 0.027	1.4 ± 0.097	0.54 ± 0.14	0.86 ± 0.21	1.5 ± 0.091	1.3 ± 0.067	0.74 ± 0.067
	TP Removed (%)	78% ± 1.9%	90% ± 2.7%	91% ± 0.40%	58% ± 2.0%	76% ± 2.0%	91% ± 3.3%	46% ± 4.1%	75% ± 6.6%	57% ± 8.8%	31% ± 4.4%	50% ± 1.9%	59% ± 3.1%
	HCP Mass (mg)	0.046 ± 0.020	0.036 ± 0.0072	0.030 ± 0.0045	0.027 ± 0.011	0.043 ± 0.0032	0.065 ± 0.0063	0.17 ± 0.033	0.13 ± 0.028	0.12 ± 0.037	0.084 ± 0.016	0.13 ± 0.017	0.14 ± 0.016
	HCP Removed (%)	69% ± 13%	70% ± 5.9%	74% ± 4.0%	77% ± 9.9%	71% ± 1.5%	33% ± 3.3%	38% ± 12.1%	48% ± 12%	46% ± 14%	64% ± 7.0%	53% ± 5.1%	30% ± 7.3%
	mAb Mass (mg)	0.22 ± 0.034	0.048 ± 0.0087	0.025 ± 0.024	0.35 ± 0.028	0.24 ± 0.023	0.075 ± 0.011	1.1 ± 0.26	0.37 ± 0.052	0.60 ± 0.21	1.2 ± 0.16	1.2 ± 0.22	0.61 ± 0.079
	mAb Removed (%)	81% ± 3.0%	95% ± 1.0%	98% ± 2.2%	72% ± 1.8%	85% ± 1.5%	93% ± 1.1%	51% ± 12%	76% ± 3.3%	72% ± 9.0%	52% ± 6.5%	59% ± 8.0%	73% ± 3.2%
	HCP TBR	0.85 ± 0.19	0.74 ± 0.085	0.76 ± 0.058	1.1 ± 0.13	0.84 ± 0.028	0.36 ± 0.10	0.76 ± 0.39	0.63 ± 0.26	0.65 ± 0.33	1.2 ± 0.17	0.89 ± 0.17	0.42 ± 0.24
CQ	TP Mass (mg)	0.86 ± 0.063	0.56 ± 0.035	0.51 ± 0.024	0.92 ± 0.031	0.88 ± 0.023	0.54 ± 0.012	1.8 ± 0.10	1.3 ± 0.087	1.5 ± 0.13	2.0 ± 0.043	1.8 ± 0.054	1.2 ± 0.080
	TP Removed (%)	33% ± 4.3%	50% ± 3.4%	52% ± 2.3%	9% ± 3.8%	33% ± 1.8%	40% ± 1.4%	29% ± 4.0%	41% ± 3.8%	29% ± 5.8%	5% ± 1.9%	31% ± 2.5%	30% ± 4.2%
	HCP Mass (mg)	0.053 ± 0.0033	0.020 ± 0.0043	0.028 ± 0.0031	0.017 ± 0.0074	0.056 ± 0.0093	0.059 ± 0.0077	0.14 ± 0.064	0.12 ± 0.026	0.11 ± 0.031	0.09 ± 0.027	0.17 ± 0.034	0.14 ± 0.016
	HCP Removed (%)	63% ± 2.9%	84% ± 3.4%	76% ± 2.6%	85% ± 6.8%	61% ± 6.4%	40% ± 7.5%	50% ± 22%	50% ± 11%	50% ± 14%	59% ± 12%	40% ± 13%	28% ± 12%
	mAb Mass (mg)	0.72 ± 0.17	0.58 ± 0.11	0.38 ± 0.027	0.66 ± 0.10	0.80 ± 0.11	0.47 ± 0.029	1.9 ± 0.13	0.79 ± 0.37	1.1 ± 0.16	1.4 ± 0.13	1.7 ± 0.20	1.1 ± 0.31
	mAb Removed (%)	38% ± 14%	38% ± 12%	65% ± 2.6%	46% ± 8.9%	48% ± 7.5%	57% ± 2.9%	19% ± 5.5%	48% ± 24%	46% ± 7.2%	43% ± 5.3%	41% ± 7.0%	47% ± 17%
	HCP TBR	1.7 ± 0.37	2.2 ± 0.31	1.2 ± 0.052	1.9 ± 0.21	1.3 ± 0.19	0.71 ± 0.19	2.6 ± 0.54	1.0 ± 0.55	1.1 ± 0.31	1.4 ± 0.23	0.95 ± 0.36	0.60 ± 0.56
